# Fragment Screening Reveals Novel Scaffolds against Sirtuin-2-Related Protein 1 from *Trypanosoma brucei*

**DOI:** 10.1021/acsomega.4c09231

**Published:** 2024-12-27

**Authors:** Renan A. Gomes, Marcelo D. Polêto, Hugo Verli, Vitor M. Almeida, Sandro R. Marana, Andreas Bender, Bruna F. Godoi, Vinícius T. L. Rodrigues, Flavio da S. Emery, Gustavo H. G. Trossini

**Affiliations:** † Departamento de Farmácia, Faculdade de Ciências Farmacêuticas, Universidade de São Paulo, Avenue. Lineu Prestes, 580, Cidade Universitária, São Paulo, São Paulo 05508-000, Brazil; ‡ Centro de Biotecnologia, Universidade Federal do Rio Grande do Sul, Avenue Bento Gonçalves, 9500, Porto Alegre, Rio Grande do Sul 91500-970, Brazil; § Departamento de Bioquímica, Instituto de Química, Universidade de São Paulo, Avenue Prof. Lineu Prestes, 748, São Paulo, São Paulo 05508-000, Brazil; ∥ Centre for Molecular Informatics, Department of Chemistry, University of Cambridge, Cambridge CB2 1EW, U.K.; ⊥ Centre for Research and Advancement in Fragments and Molecular Targets (CRAFT), Departamento de Ciências Farmacêuticas, Faculdade de Ciências Farmacêuticas de Ribeirão Preto, Universidade de São Paulo, Av. Prof. Zeferino Vaz, Campus USP, Ribeirão Preto, São Paulo 14040-903, Brazil

## Abstract

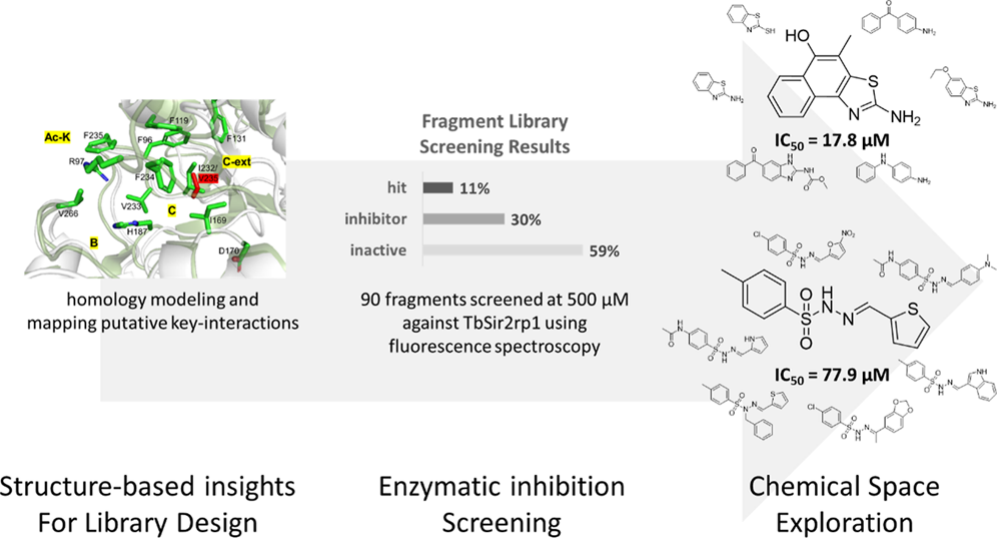

Sirtuin-2 (Sir2) is a histone deacetylase recognized as an antitrypanosomal target, yet there is limited knowledge regarding their potent inhibitors. This investigation employs the fragment-based drug discovery (FBDD) framework to identify novel inhibitors against *Trypanosoma brucei* Sir2-related protein 1. Initially, frequent residue–ligand interactions extracted from the crystallographic structures of human Sir2 and key features of human and parasitic Sir2 active sites were utilized to curate a targeted fragment library. Screening identified ten fragment hits, which introduced nine novel substructures compared to known Sir2 inhibitors. Among these, fragment 1 was the most potent, with an IC_50_ value of 17.8 μM and a ligand efficiency of 0.41. Further chemical space exploration of 30 compounds from the two most promising hits confirmed fragment 1 as the most potent. This study underscores the effectiveness of FBDD in discovering chemically distinct starting points with favorable ligand efficiency against protein targets in infectious diseases.

## Introduction

**Figure 1:**
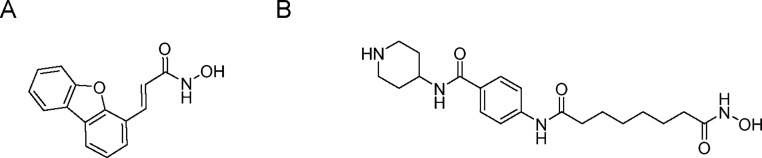
Compounds TB56 (A) and procaine–SAHA derivative (B) reported as inhibitors of epigenetic targets with *in vivo* efficacy.

Trypanosomatids are parasitic organisms that require rapid adaptation to varying environmental stimuli, including evasion of host immune responses and drug interventions.^1−4^ To survive these challenges, they employ sophisticated epigenetic regulatory mechanisms that involve structural modifications of genetic material, such as alkylation or acylation, without altering the nucleotide order or quantity. These modifications influence transcription, replication, and gene repair processes.^5^ Due to their critical role in parasite survival, epigenetic regulatory proteins have emerged as potential targets for new antiparasitic drugs. Although no epigenetic inhibitors have reached clinical use, several have shown an *in vivo* activity. For instance, the tcDAC2 (*Trypanosoma cruzi* deacetylase 2) inhibitor TB56 (Figure 1A) reduced parasite burden in *T. cruzi*-infected mice,^6^ while a compound (Figure 1B) combining SAHA and procainamide (a histone deacetylase inhibitor and a DNA methyltransferase inhibitor, respectively) showed efficacy in severe malaria models.^7^

The sirtuin-2-related protein 1 (TbSir2rp1) was selected in this work as an epigenetic regulatory target in *T. brucei*, the causative agent of sleeping sickness.^8^ Sir2 enzymes are NAD^+^-dependent lysine deacetylases (class III KDACs/HDACs) found across diverse organisms, from bacteria to humans.^9^ They share conserved structural features, including the Rossmann fold for NAD^+^ binding, Zn^2+^-binding cysteines, and the catalytic site.^10−12^ Sir2 enzymes catalyze the removal of acetyl groups from lysine residues in histones H2A and H2B using NAD^+^ as a cosubstrate,^13,14^ with evidence suggesting that acetylated lysine binds first, followed by NAD^+^, to form the catalytically active complex.^15,16^ Also, due to its critical role in *T. brucei*, particularly in DNA damage response, this NAD-dependent enzyme catalyzes the ADP-ribosylation of these histones, impacting chromatin structure and cellular resistance to DNA damage, as demonstrated by García-Salcedo et al.^16^ This distinct function highlights its potential as a drug target.

**Figure 2:**
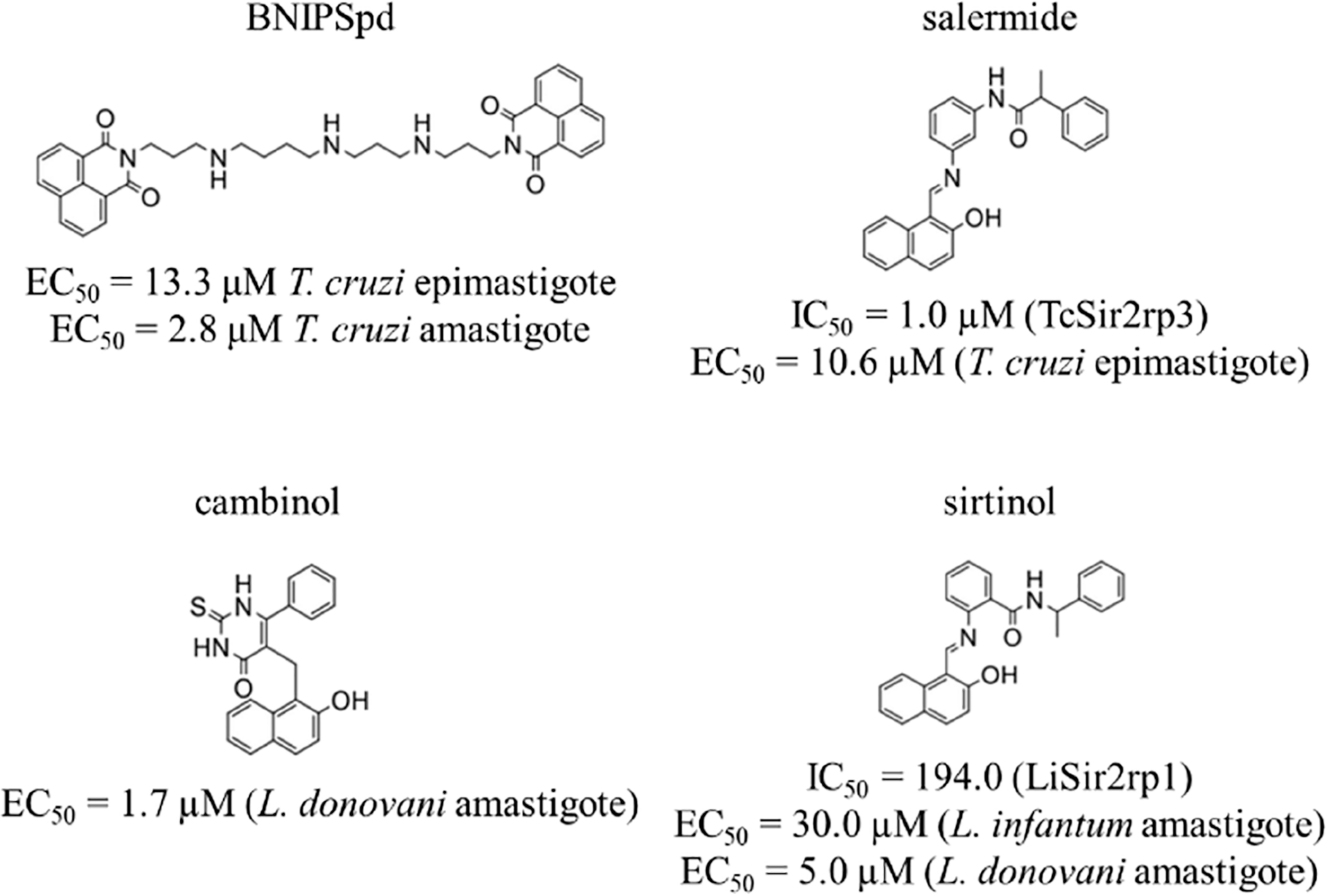
Sir2 inhibitors and respective *in vitro* activities.

Human Sir2 (hSir2) enzymes are implicated in tumor development, leading several hSir2 inhibitors previously used in campaigns for the development of new antineoplastic drugs (e.g., salermide, bisnaphthalimidopropyl (BNIPs), sirtinol, and cambinol in Figure 2) to be tested as inhibitors of trypanosomatid Sir2, such as *T. cruzi* Sir2rp3, given the 27% identity with human Sir2, or *Leishmania infantum* Sir2rp1, 42% identity (sequences are available in Supporting Information).^17−23^ Despite evolutionary conservation of sirtuins, these inhibitors show limited potency and selectivity against parasitic Sir2, with micromolar IC_50_/EC_50_ values and low selectivity indices,^19,24^ hindering their effectiveness as antiparasitic candidates.

In this study, we employed a fragment-based drug design (FBDD) approach to discover novel inhibitors for sirtuin 2-related protein 1 (TbSir2rp1). A fragment library was constructed to target the active site properties of Sir2, based on the structural features of the binding site of TbSir2rp1 and human Sir2 (hSir2). Residues that frequently interact with hSir2 cocrystallized ligands were identified, leveraging the conserved catalytic site. Additionally, residues defined as “hotspots” with potential for strong target–ligand interactions were identified. Fragment screening against TbSir2rp1 was performed via fluorescence spectroscopy, and the best hits’ IC_50_ values were determined. Results confirmed our hypothesis: hydrophobic fragments showed greater affinity for the enzyme’s binding site, introducing nine novel substructures compared to known hSir2 inhibitors. Exploration of the chemical space around the top fragments revealed that fragment 1 remained the most potent.

## Methods

### Target Modeling

The TbSir2rp1 sequence was obtained from the NCBI database.^25^ SignalP 4.0^26^ was used for preprocessing to identify potential signal peptides. Structural models of Sir2rp1 were generated using the RaptorX server,^27^ selecting templates with sequence identity above 30%, calculated via EMBOSS Needle.^28^ Sequence alignments with templates were performed using Expresso.^29^ The best Sir2rp1 model was selected based on stereochemical evaluation using QMEAN^30^ and WHATCHECK,^31^ complemented by secondary structure and disorder region analysis. Sequences were assessed for disorder regions using DIsEMBL,^32^ GLOBPLOT,^33^ PONDR,^34^ MESSA,^35^ MFDp2,^36^ and DISOPRED^37^ servers.

#### Binding Site Analysis

For key residue identification, hSir2 structures in the PDB with substrates or inhibitors were analyzed using Discovery Studio Visualizer,^38^ transferring the knowledge about the catalytic site of hSir2 to the parasitic enzyme, since the sites are known to be highly conserved.^11^ Tertiary structures of hSir2 and TbSir2rp1 were submitted to FTMap.^39^ Through extensive sampling and scoring of billions of poses, employing 16 small organic molecules as probes, this mapping server identifies surface regions making significant contributions to the ligand binding free energy (i.e., hotspots). Regions with at least 16 probes were considered to be hotspots.

### Fragment Library Construction

For the selection of the molecular fragments, comprising both in-house and commercial compounds that would be part of the initial library, the rule of three (Ro3) was used as a reference–compounds with molecular weight (MW) < 300 Da, calculated logarithm of the 1-octanol–water partition coefficient of the nonionized molecule (cLogP) ≤ 3, number of hydrogen bond donors (HBDs) ≤ 3, number of hydrogen bond acceptors (HBAs) ≤ 3, and number of rotatable bonds (nRot) ≤ 3.^40^ The only rule consistently followed for all compounds was the molecular weight (MW), whereas violations of other criteria were allowed to broaden the assessment of chemical space.^41,42^

#### Protein Production

The following method was based on Moretti et al.^17^ and Kowieski et al.^43^ The TbSir2rp1 gene was cloned into the pET24(+) expression plasmid (Novagen), which facilitates purification by providing a histidine tag and kanamycin resistance. The plasmid (DNA sequence of the expressed TbSir2rp1 in the Supporting Information) was then transformed into *Escherichia coli* BL21-Codon Plus (DE3)-RIL strain via heat shock (30 min on ice, followed by 40 s at 42 °C and then 5 min on ice). Following transformation, bacteria were plated on Luria–Bertani (LB) agar containing 50 μg/mL kanamycin and incubated at 37 °C for 18 h. A single colony was picked and inoculated into 5 mL of LB medium with kanamycin and grown at 37 °C with shaking for 18 h. The preculture was then added to 500 mL of LB medium and incubated at 37 °C with shaking until reaching an optical density at 600 nm (OD600) of 0.7. Recombinant TbSir2rp1 expression was induced with 0.5 mM IPTG (isopropyl β-*D*-thiogalactoside) for 18 h at 25 °C. After induction, the culture was centrifuged for 20 min to remove the culture medium. The bacterial pellet was resuspended in lysis buffer (200 mM NaCl, 5% glycerol, 5 mM 2-mercaptoethanol in 25 mM HEPES buffer, pH 7.5) and lysed using a Branson Sonifier 250 (Branson Instruments, Stanford, USA) with four 12 s pulses at 30% output. The soluble fraction was separated by centrifugation and incubated with Ni-NTA resin for 30 min. The resin was washed with lysis buffer containing 20 mM imidazole, followed by washing with 40 and 60 mM imidazole. The purified protein was eluted with lysis buffer containing 300 mM imidazole.

#### Inhibitory Assays

The concentration of the acetylated peptide substrate, NAD^+^, and the incubation time were optimized to maximize the use of enzymes and substrates (results in Figure S1). The acetylated peptide corresponds to the sequence Abz-Gly-Pro-acetylLys-SerGln–EDDnp, where Abz is ortho-aminobenzoic acid and EDDnp is *N*-[2,4-dinitrophenyl]ethylenediamine.

To halt the deacetylation reaction, 4 mM nicotinamide was used. For each pair of wells in the 96-well plate, 0.2 mg/mL of trypsin was added to only one well to hydrolyze the acetylated peptide, enabling fluorescence emission. Fluorescence was measured immediately at 420 nm with excitation at 320 nm using a SpectraMax M2 microplate reader (Molecular Devices).

Deacetylation activity was determined by the difference between the well where trypsin was added and the well without trypsin. All assays were performed in triplicate, and activity values were reported as means ± standard deviations with outliers identified and excluded from the calculations. Statistical analyses were conducted using Prism 7 software (GraphPad).

The screening of the 90 fragments was performed at a single concentration of 500 μM in triplicate. The enzyme was used at 1 μM, and the substrates NAD^+^ and acetylated peptide were used at 100 and 50 μM, respectively. Incubation was carried out at 37.0 °C for 2 h. Each fragment was added to a pair of wells, with trypsin being added to only one well, as previously described for inhibitory activity calculation. The inhibition results were classified as “inactive” if the *p*-value in the unpaired *t*-test with Welch’s correction relative to the negative control was greater than 0.05; as “inhibitor” if the *p*-value was less than or equal to 0.05 and the inhibitory activity was below 50%; and as “hit” if the *p*-value was less than or equal to 0.05 and the inhibitory activity was greater than or equal to 50%.

Fragments with higher potential to proceed to the next phase of the study, based on potency or synthetic versatility, had their IC_50_ values determined under the same experimental conditions, with the inhibitor concentration being varied. Ligand efficiency (LE) was calculated as LE = –*R*·*T*·ln(IC_50_)/HAC using IC_50_ values instead of *K*_D_ for comparison. Promising fragments underwent initial optimization involving structurally similar compounds. IC_50_ was determined for successful compounds. The inhibition mechanism was characterized using Lineweaver–Burk plots under five concentrations of each substrate and three concentrations of the fragment. IC_50_ values were determined with at least seven different concentrations, and Lineweaver–Burk analysis used six concentrations.

### Chemical Space Analysis

Chemical descriptors were calculated using RDKit,^44^ and the statistical analyses were performed using Prism 7 software (GraphPad). The scaffold analysis was performed using the ScaffoldGraph Python library.

#### Docking

Molecular docking was performed using Glide.^45^ The receptor box dimensions selected for docking were sufficiently large to encompass the entire binding site of the enzyme. Ligands underwent ionization and low-energy ring conformation generation with LigPrep while preserving the stereochemistry. The extra precision (XP) mode with default parameters was employed as a quantitative criterion to evaluate the poses generated for each molecular fragment. The pose generation algorithm continued to produce new poses only if the ranking of the new pose differed significantly from that of the previously generated pose. Protein structure was then minimized with the OPLS-2005 force field.

## Results and Discussion

### Analysis of the Target Binding Site

**Figure 3:**
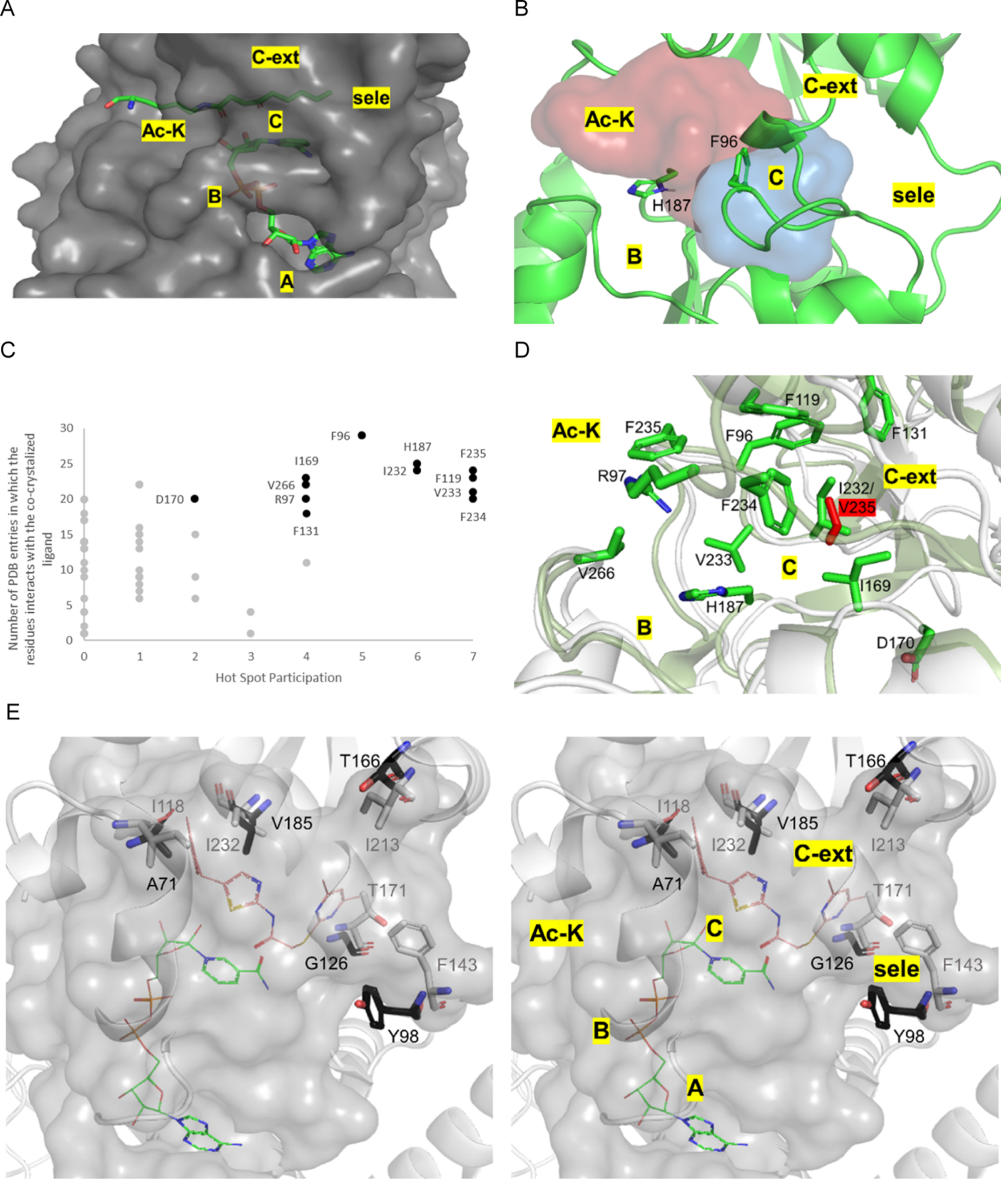
(A) Human Sir2 structure (surface) and both substrates (in sticks), acylated lysine and NAD^+^. Subdivisions of the sirtuin-2 binding site: A—entrance for NAD^+^, B—phosphate interaction site, C—catalysis subregion, C-ext—subregion opened after both substrates binding, Ac–K—entrance for the acetylated lysine of the peptide substrate, and sele—subregion that provides selectivity against other human sirtuins. (B) Hotspots in the Ac–K (red surface) and C (blue surface) subregions of the catalytic site. This suggests that novel ligands will likely compete with at least one of the natural substrates, either acetylated peptide or NAD^+^. (C) In black, residues from the catalytic site selected based on their high frequency of interaction with cocrystallized ligands in PDB entries, as well as instances where they were part of hotspots. In light gray, remaining catalytic site residues that were not selected. (D) Distribution of 12 residues (sticks) across the Sir2 binding site, both human (green) and trypanosomatid (white) Sir2; the only divergent residue in TbSir2rp1 is highlighted in red. (E) Parasitic residues in C-ext and selectivity pockets (A71, V185, G126, T166, and Y98) offer potential for selective inhibitor design through bulky, polar, or HBA/HBD group additions; NAD^+^ (green wires) and inhibitor (pink wires) from PDB 4RMG for reference. Parasitic residues in black and human in gray.

To identify inhibitors of TbSir2rp1, we adopted a fragment-based drug design (FBDD) approach. Over the past 20 years, few studies have utilized this approach for other targets of this trypanosome.^46−48^ Given the relatively limited chemical space assessed in a screening campaign, libraries composed of molecular fragments offer the opportunity to assess advantageous starting points compared to approaches utilizing larger and structurally complex compounds.^49,50^ Furthermore, given the absence of information derived from X-ray crystallography or cryo-EM and considering that the establishment of a fragment library demands less insight into the target compared to libraries composed of conventional drug-like compounds, the development of the initial fragment library emerges as an ideal approach.^51^ For the construction of a fragment library with chemical characteristics focused on the active site of Sir2 properties, an initial analysis of their binding sites was carried out. The structure of TbSir2rp1 was modeled using the RaptorX server^27^ and subsequently successfully assessed using different tools for evaluating secondary and tertiary structures (Table S1). We identified the residues that (1) frequently interact with ligands cocrystallized with hSir2 and (2) are “hotspots” aiming to optimize ligand efficiency. For this characterization, the catalytic site was divided into six subregions as proposed by Swyter et al.^52^ (Figure 3A). Notably, residues F96 and H187 interacted with various ligands in 29 and 25 complexes out of 29, respectively, likely due to their central positions in the interface of subregions Ac–K, B, and C. Of the nine most frequently interacting residues, eight were nonpolar and four aromatic, suggesting a predominantly hydrophobic cavity. Additionally, seven of the top 18 residues were phenylalanines.

Next, FTMap was used to map the hotspots in the binding site. The standard practice in the literature recommends using only the apo form of proteins to prevent hotspots from being defined based on conformational changes induced by specific ligands.^39^ However, both complexed and apo structures were included, given that Sir2’s catalytic site undergoes various ligand-induced conformational shifts. In the apo state, the site ranges from an open to a partially closed form (with one substrate bound), a fully closed ternary complex, or a SirReal-inhibitor-induced closed variant.^52^ Representative structures of these conformations were analyzed to determine how ligand-induced changes could affect hotspot exposure. Across conformations, probes clustered in Ac–K and C subregions (Figure 3B), indicating that new ligands are likely to compete with at least one substrate (either acetylated peptide or NAD^+^), despite the site’s flexibility to accommodate small molecules beyond these endogenous substrates. Also, of the seven most representative amino acids (Figure 3C), six were apolar and five were aromatic, emphasizing the importance of these characteristics in ligand interactions with the catalytic site, as highlighted in the previous analysis.

The selection of active site key residues comprised a total of 12 amino acids, namely (numbering according to hSIR2): H187, V233, F235, and V266 in the subregion corresponding to the binding site for the acetylated lysine (Ac–K); F96, R97, I169, and D170 in the nicotinamide subregion (in C) (both interact with the amide moiety of nicotinamide via hydrogen bonds as observed in the hSir2 PDB entry 4RMG); and F119, F131, I232 (valine in trypanosomatid Sir2), and F234 in the extension of the nicotinamide subregion (in C-ext). In all evaluated sequences of hSir2 and TbSir2rp1, the 12 selected residues were consistently observed in identical positions with the exception of I232 and V235, which were exclusively present in the human and parasitic enzymes, respectively.

To evaluate the conservation of catalytic sites between hSir2 and TbSir2rp1, we first aligned their sequences (Supporting Information). Among the 12 consensus residues analyzed, 11 were identical between the human and parasitic proteins, with only one residue, V185 in Sir2rp1, differing from the corresponding I232 in human hSir2. Both residues feature nonpolar, aliphatic side chains yet differ in volume: the isopropyl group of valine is smaller than the *sec*-butyl group of isoleucine. This steric difference presents an opportunity to enhance the selectivity for parasitic enzyme inhibition by targeting this region. As a complementary approach, we aligned 12 sequences of TbSir2rp1 obtained from the TriTrypDB, Uniprot, and NCBI databases, confirming no variation in any of the 12 conserved residues.

To identify residues with the potential to improve selectivity, we expanded our comparison to include all residues interacting with ligands, focusing on side chains with contrasting properties (size, polarity, and p*K*_a_) in equivalent positions, rather than those involved in hotspots or frequent ligand interactions. We identified 17 divergent residues between the human and *T. brucei* enzymes. To avoid potential off-target effects, all residues in subregions A and B, which interact with the ADP-ribose moiety of NAD^+^, were excluded from further consideration as developing inhibitors mimicking NAD^+^ structure could lead to broad and undesirable biological activity. Within the C-ext subregion (Figure 3E), parasitic residues A71 and V185 correspond to human I118 and I232, respectively, with smaller side chains that support the addition of bulky groups during fragment growth. In the selectivity pocket, parasitic G126, equivalent to human T171, similarly supports larger fragment addition. The presence of T166 in TbSir2rp1, corresponding to human I213, permits bulky, polar, and HBA/HBD groups, offering a favorable target for selective inhibition. Finally, Y98 in TbSir2rp1, diverging from human F143 in the selectivity pocket, further supports the use of polar groups or HBA/HBD functionalities. Therefore, five residues present opportunities for enhancing inhibitor selectivity.

### Construction of the Fragment Library

In FBDD, selecting fragments based on functional rather than structural diversity improves the interaction variety and enhances information recovery with novel targets. Functionally diverse libraries, particularly those optimized for specific interactions, yield more target-specific information than structurally varied libraries.^53^ Fragments that often succeed in initial drug discovery campaigns balance polar functionality and hydrophobicity, leveraging enthalpy-driven hydrogen bonding and entropy-driven dispersion forces that favor binding through complementarity to hydrophobic pockets and desolvation.^51^ Since binding site analysis revealed a predominantly hydrophobic cavity with a preference for nonpolar and aromatic interactions, this insight was incorporated into the fragment selection criteria. We chose fragments under 300 Da, prioritizing the rule of three (≤3 rotatable bonds, 3 H-bond donors/acceptors, and logP ≤3) and incorporating positive logP values and at least one aromatic ring. This led to the selection of 90 fragments to form the focused library.

### Fragment Screening

The fragment library was screened against TbSir2rp1 with an initial concentration of 500 μM using fluorescence spectroscopy. In FBDD, orthogonal validation enhances hit confirmation across all screened fragments, including low-affinity targets and those with PAINS motifs. However, studies often rely on a single verification method.^54,55^ The screening identified 40 fragments with inhibitory activity against TbSir2rp1, but no orthogonal validation was performed. The fragment hits (i.e., at least 50% inhibition at the screening concentration) are indicated in Table 1 (a comprehensive list of results is provided in Table S1). Fragment #11 displayed significant variability in results (inhibition of 88.7 ± 31.3%); #12 and #13 have moieties reported as pan assay interference compounds (PAINs), a Michael acceptor and a quinone derivative, respectively. Consequently, only fragments 1–10 were deemed as hits (11% of the hit rate). Typical hit rates for fragment screening campaigns are generally up to 5% for techniques that do not determine modes of binding (e.g., surface plasmon resonance and affinity capillary electrophoresis),^56^ and the 11% hit rate observed here suggests that targeted libraries can yield a greater number of hits compared to a library strictly adhering to the Ro3.

**Table 1:**
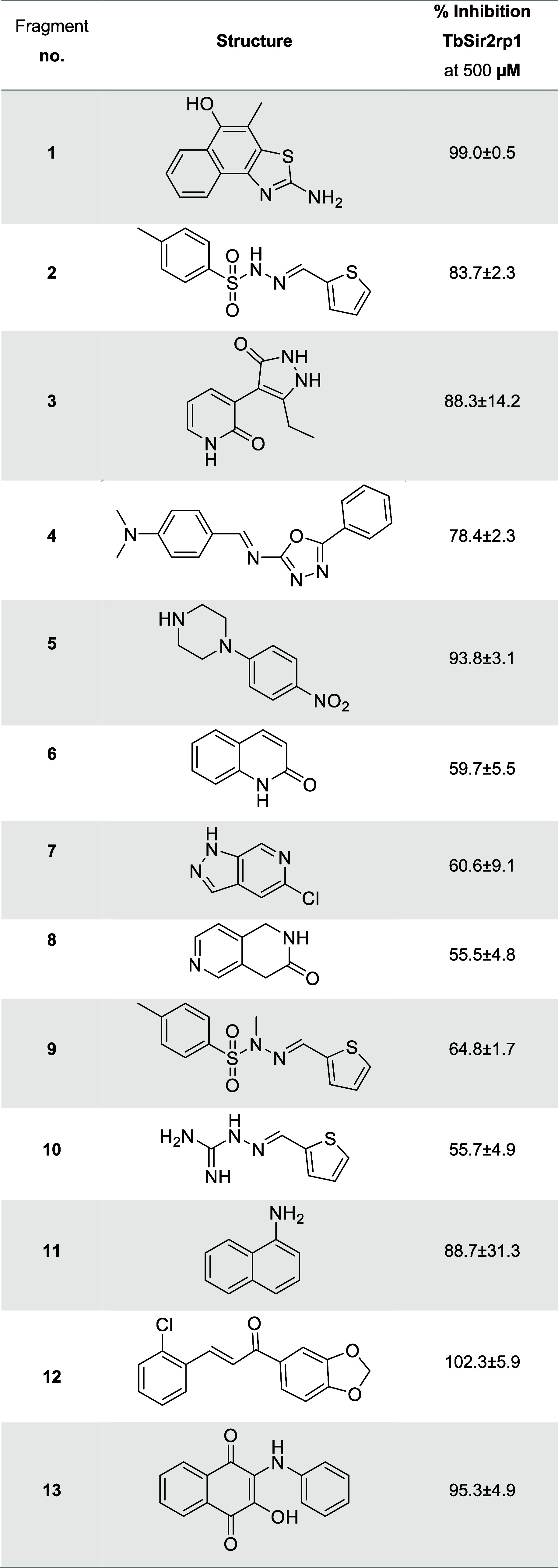
Fragment-Based Screening Results Using a Fluorescence Spectroscopy Readout against TbSir2rp1

**Figure 4:**
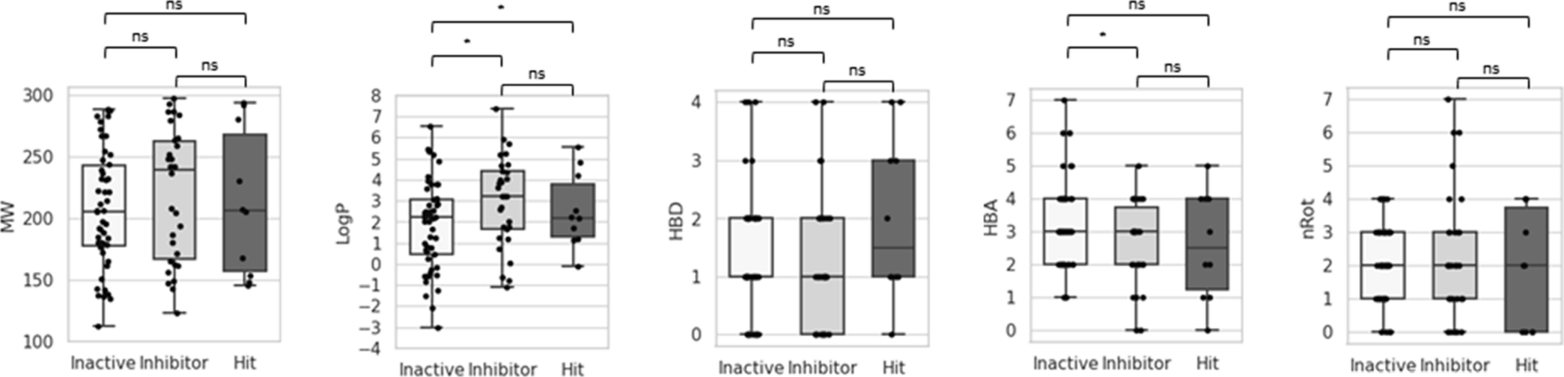
Distribution of molecular descriptors for the screened library, categorized by the activity level: “inactive” for compounds with no significant inhibition (paired *t*-test), “inhibitor” for significant inhibition, and “hit” for inhibition equal to or greater than 50%. Descriptors include molecular weight (MW), 1-octanol–water partition coefficient (cLogP), number of rotatable bonds (nRot), and number of hydrogen bond acceptors (HBA) and donors (HBD).

Considering the chemical properties (Figure 4), as expected, fragments with logP values lower than 1.0 were more commonly found among the inactive compounds (Welch *t*-test *p*-value = 0.02) since most of the key residues at the binding site are hydrophobic. The number of HBA was significantly lower in active compounds (Welch *t*-test *p*-value = 0.03), which reinforces the affinity between hydrophobic fragments and the TbSir2rp1 binding site. Furthermore, six out of ten hits were discovered to exceed the Ro3 threshold. Fragments #2, #4, and #9 exhibited three violations of Ro3 criteria (each possessing high clogP and nRot values), while fragments #3, #5, and #10 exhibited only one violation (all having four hydrogen bond acceptor groups).

**Figure 5:**
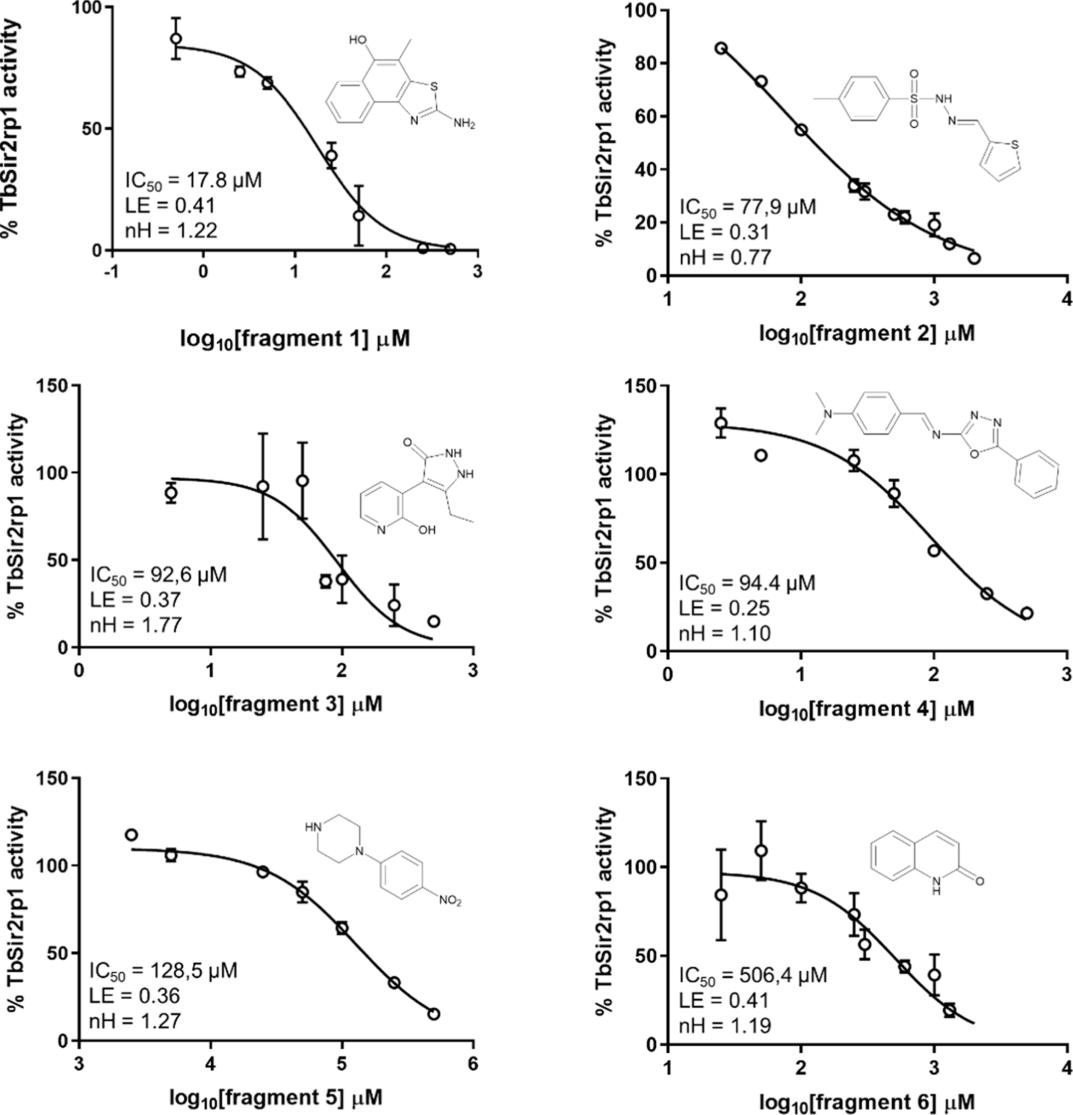
Concentration–response curves for fragments 1–6 demonstrate their activity against TbSir2rp1. Chemical structure, IC_50_ values, Hill coefficients (nHill), and ligand efficiency (LE) for each fragment are shown.

As the trio composed of fragments #2–#9–#10 presented a highly similar structure, the most potent and easiest to obtain fragment (#2) was chosen for IC_50_ determination (Figure 5). Similarly, among the remaining group with comparable inhibitory activity (fragments #6, #7, and #8), hit #6 was selected for its ease of availability. Furthermore, IC_50_ values were obtained for fragments #1 through #6. Four of these six fragments proved to be potent with their respective IC_50_ values below 100 μM, and five of them showed a high relationship between potency and the number of heavy atoms as they presented LE values above 0.30. Fragment 1 was the most notable for having the highest LE value, along with fragment 6, and it was the most potent in the library with an IC_50_ of 17.8 μM.

**Figure 6:**
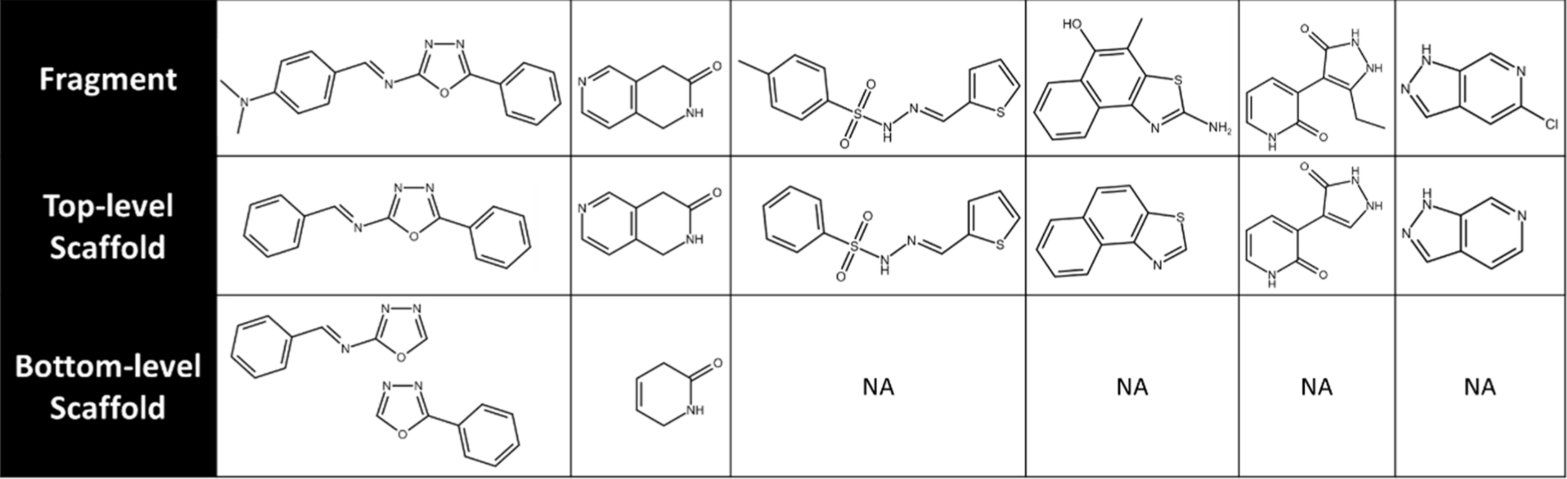
Hits’ scaffolds not present in the structure of already reported human sirtuin-2 (hSir2) inhibitors. The top-level scaffold represents the ring systems and their linkers, while the bottom level represents the subsequent removal of a peripheral ring and the possibly resulting side chains. NA: not applicable, indicating the scaffolds/substructures obtained upon further simplification were present in already reported hSir2 inhibitors.

Out of the ten hits targeting the parasitic Sir2, nine distinct molecular scaffolds were identified since fragments #2 and #9 shared the same scaffold. Upon decomposing the hits into substructures, six top-level scaffolds and three bottom-level scaffolds did not correspond to any substructure of the 2395 compounds present in the ChEMBL33^57^ database with reported inhibitory activity against hSir2 (Figure 6). It is important to note that this discovery is based on inhibitors of hSir2, which possesses a highly conserved catalytic site in comparison to TbSir2rp1, as previously discussed. These novel substructures corroborate the innovative capacity of the FBDD framework to be used for the design of new enzymatic inhibitors. Fragments 1 and 2 were selected for an initial optimization study.

### SAR Analysis of Fragments 1 and 2

**Figure 7:**
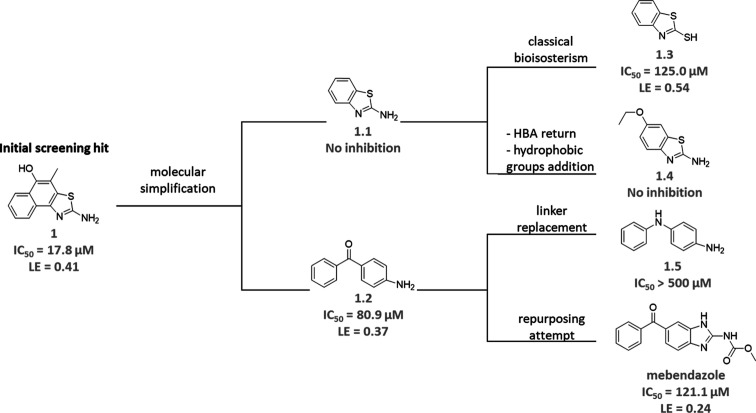
SAR exploration of fragment 1. HBA, hydrogen bond acceptor group.

Fragment 1 was selected for its potency, high LE, and previously reported trypanocidal activity against *T. brucei*, showing a minimum lytic concentration of 100 μM and a maximum tolerated dose of 50 mg/kg in infected mice.^58^ Our results provide insights into its mode of action; however, its low structural complexity suggests that it probably interacts with additional parasitic targets. As an initial approach to enhance the potency of fragment 1, molecular simplification was applied, yielding two new compounds: fragments 1.1 and 1.2 (Figure 7). While the former did not inhibit TbSir2rp1, the latter exhibited an IC_50_ of 80.9 μM (nH = 0.72) and an LE of 0.37. From 1.1, two modifications were made: a classical bioisosterism by replacing the amino group with the mercapto group, resulting in fragment 1.3 with IC_50_ = 125.0 μM (nH = 1.04) and LE = 0.54, and the return of the hydrogen bond acceptor group, but with the addition of the hydrophobic ethyl group, resulting in fragment 1.4, which did not inhibit TbSir2rp1. From 1.2, fragment 1.5 was obtained by the replacement of the linker group by a secondary amine, which had an IC_50_ above 500 μM. Finally, looking for repurposing opportunities, 1.2 was used in substructure-based screening, which resulted in the anthelmintic drug mebendazole with an IC_50_ of 121.1 μM (nH = 1.25) and LE = 0.24, potency very close to 1.3.

Although derivatives of fragment 1 showed lower potency, fragment 1.2 offers improved accessibility and greater synthetic versatility, while fragment 1.3 demonstrates significantly higher ligand efficiency (LE), making both promising candidates for future investigation. As expected, more complex compounds than fragment 1 led to reductions in LE, as observed in the comparison between 1.2 and mebendazole (reduction of LE from 0.37 to 0.24). The substituent groups with hydrogen-bond acceptors were present in the most potent compounds, 1, 1.2, and mebendazole. In 1.4, the presence of the additional hydrophobic ethyl group had a detrimental effect on inhibitory activity.

**Figure 8:**
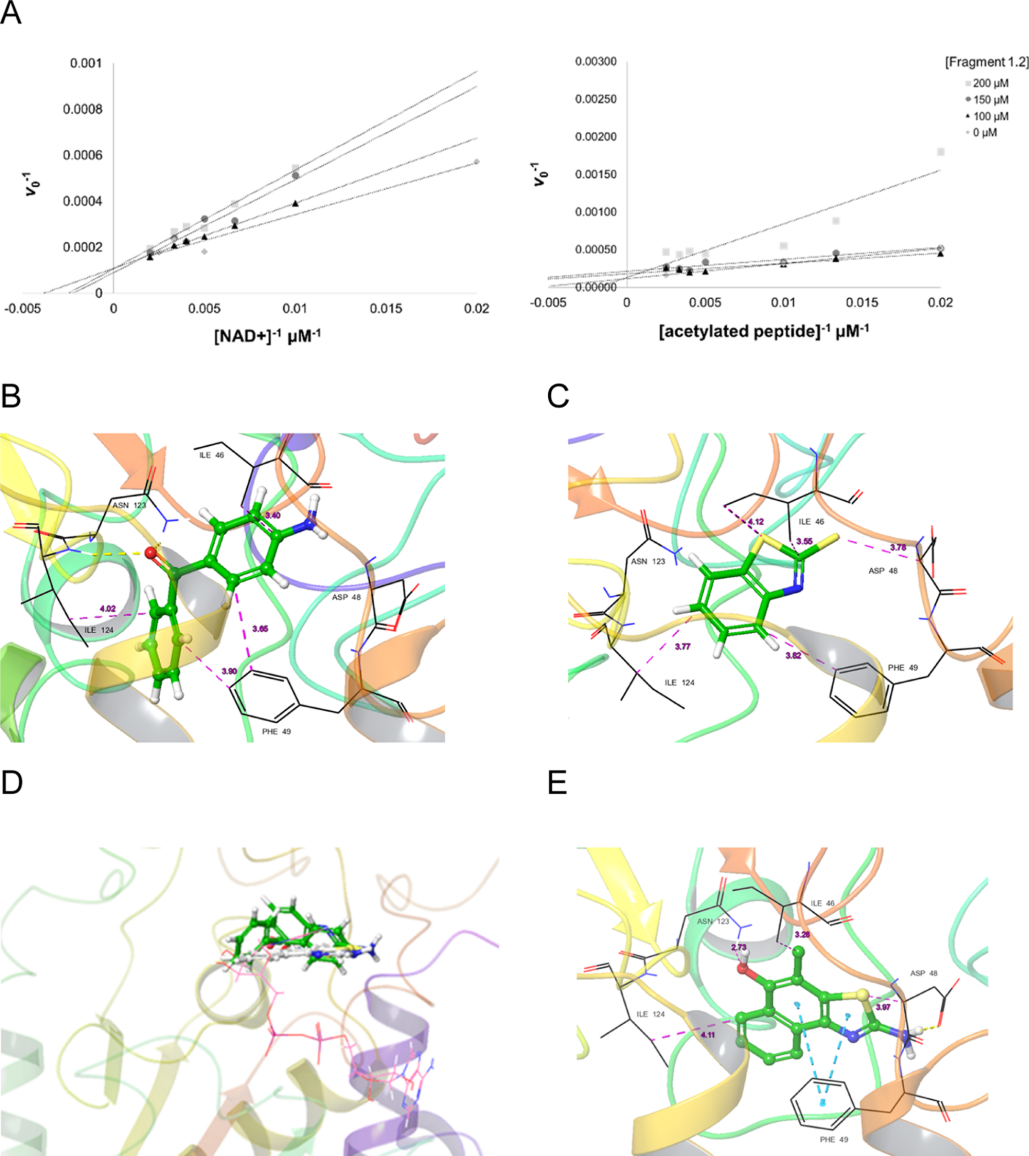
(A) Lineweaver–Burk plots for fragment 1.2 in relation to the acetylated peptide substrate and NAD^+^. Prediction of binding modes in TbSir2rp1 by molecular docking of fragments 1.2 (B), 1.3 (C), all fragments overlaid and NAD^+^ superimposed as reference (D), and 1 (E), which possesses a comparable structure to theirs superimposed and interacts similarly.

As the final step of the initial phase of enzymatic inhibition assays, the inhibition mechanisms of 1.2 against both substrates were determined. In the Lineweaver–Burk plot (Figure 8A), the unchanged value of *V*_max_, despite variations in inhibitor concentration, characterized the inhibition mechanism as competitive for both substrates, acetylated peptide and NAD^+^. Based on these results and the division of the catalytic site, it was concluded that the residues interacting with 1.2 would be situated at the interface of the C subregion. To investigate this new hypothesis, a binding mode prediction study was conducted to rationalize the experimentally determined potency values for fragments 1.2 and 1.3, as well as for 1. Fragments 1.2 and 1.3 were derived from the simplification of fragment 1, retaining key substructures without being identical. For instance, the primary amine attached to an aromatic ring was preserved exclusively in fragment 1.2, while the benzothiazole group was retained only in fragment 1.3. Consequently, molecular docking of these three fragments was performed on the previously established TbSir2rp1 model to assess whether the interactions observed with fragment 1 would encompass those of fragments 1.2 and 1.3 collectively, as expected from the FBDD merging approach. The generated poses for the two simpler fragments (Figure 8B,C) revealed interactions with the previously identified key residues in C subregion F49 and I124, which are equivalent to F96 and I169 in hSir2, respectively. The overlay of their binding modes (Figure 8D) matched the predicted binding mode for 1 (Figure 8E), supporting the proposed assumption. Additionally, the predicted interactions in the binding modes were consistent with the observed potency in *in vitro* assays. Fragment 1.3 exhibited a lower IC_50_ and interacted with five residues in the catalytic site via nonpolar contacts. Fragment 1.2 showed an intermediate IC_50_ and interacted with six residues, including two hydrogen bonds. Lastly, fragment 1 was the most potent, establishing three nonpolar contacts, two π interactions, and two hydrogen bonds.

Fragment 2, which had an IC_50_ below 100 μM, could be conveniently synthesized in two steps and showed low cytotoxicity *in vitro* against human lung cells (WI-26VA-4) with a CC_50_ of 226 μM (result previously published by our group).^59^ The exploration of the chemical space around fragment 2 was based on maintaining the sulfonyl–hydrazone moiety while varying the groups linked to it, originally the 4-methylphenyl and thien-2-yl groups. This 22-compound library was previously acquired in another study conducted by our group.^59^ The compounds to be tested were selected based on modifications in three different regions: (I) replacement of the methyl group at position 4 on the benzene ring by groups with different size and polarity, (II) substitution of the aromatic system linked to the imine group, and (III) the replacement of the acidic hydrogen on the sulfonyl–hydrazone group by a hydrophobic group.

Twenty-two compounds were tested at a single concentration of 100 μM against TbSir2rp1 (in Supporting Information), and none of them were found to be more potent than fragment 2; hence, they were not selected for further assessment. Among the relatively most potent compounds in the series, there was no apparent consensus regarding which substitutions in region I positively contributed to the inhibitory activity of the compounds. This is possibly due to the hydrophilic ethanamide group and the hydrophobic methyl and chloride groups not being close enough to any region of the cavity to establish interactions. This suggests that this modification point in fragment 2 could be used for the introduction of a spacer group. Substitutions in region II found in the most potent analogues converged toward aromatic heterocyclic groups with five or six atoms (thiophene, furan, pyrrole, and benzene), in some analogues substituted with hydrogen-bond acceptors. This indicated that larger substituents at this position might not be suitable, such as benzo[*d*][1,3]dioxol-5-yl and 1H-indol-3-yl (see the Supporting Information section, Table S3, green substructure in compounds 2.15 and 2.17, respectively). In region III, the sole modification evaluated, the addition of a benzyl group, did not significantly impact the potency of the analogue as it remained close to the 100 μM threshold. Additionally, the antimicrobial drugs sulfamethazine and sulfamethoxazole were evaluated for their possible repurposing potential. While the former did not show consistency and/or reproducibility in tests, the latter did not induce any inhibition of TbSir2rp1, and hence, this approach was not successful.

This study employed a fragment-based drug discovery approach to identify novel inhibitors of sirtuin-2-related protein 1 from *T. brucei*. The selected fragments exhibited promising inhibitory activities covering nine novel substructures, with compound 1 standing out as the most potent, at IC_50_ = 17.8 μM. SAR exploration of compound 1 revealed that structural simplifications were possible with a low impact on potency. The Lineweaver–Burk analysis provided insights into the competitive inhibition mechanism of compounds 1.2 and, by extension, 1 due to their similar structure and potency. Additionally, the exploration of chemical space around fragment 2 (IC_50_ = 77.9 μM) revealed challenges in identifying potent analogues, suggesting a potential modification point for introducing spacer groups. This work hence underscores the potential of FBDD for designing enzyme inhibitors and opens avenues for the further development of antiparasitic drugs targeting trypanosomatid Sir2.

## Conclusions

This study highlights the effectiveness of the FBDD approach in identifying novel inhibitors of TbSir2rp1. By leveraging frequent residue–ligand interactions from crystallographic structures of human Sir2 and analyzing conserved features of Sir2 active sites, a targeted fragment library was curated. Screening efforts identified ten promising hits, introducing nine novel substructures previously unreported for Sir2 inhibition. Notably, fragment 1 emerged as the most potent, exhibiting an IC_50_ value of 17.8 μM and a ligand efficiency of 0.41. The subsequent exploration of the chemical space surrounding the two most promising hits further validated fragment 1 as the most effective candidate. This work not only provides new insights into inhibitor development for epigenetic targets in parasitic diseases but also demonstrates the utility of FBDD in discovering chemically diverse effective starting points for drug development.

## Supporting Information Available

Sequence alignment of human Sir2 and TbSir2rp1 in the file “emboss_needle_alignment_hSir2_TbSir2rp1.out”; DNA sequence of the expressed TbSir2rp1; stereochemical evaluation of the TbSir2rp1 model; fragment library details and screening results; results of fragment 2 derivatives; and screening of fluorometric assay conditions (PDF)
