# Supplementary material for: Fragment Screening Reveals Novel Scaffolds against Sirtuin-2-Related Protein 1 from Trypanosoma brucei
Source: ACS Omega. 2024 Dec 27;10(4):3808–19. doi: 10.1021/acsomega.4c09231 (PMC11799985; doi:10.1021/acsomega.4c09231)

Supporting Information

Title

Fragment-screening reveal novel scaffolds against Sirtuin-2 related protein 1 from *Trypanosoma brucei*

Authors

Renan A. Gomes[a], Marcelo D. Polêto[b], Hugo Verli[b], Vitor M. Almeida[c], Sandro R. Marana[c], Andreas Bender[d], Bruna F. Godoi[e]; Vinícius Rodrigues[e], Flavio da S. Emery[e], Gustavo H. G. Trossini[a]*

Email: trossini@usp.br

Affiliations

[a] Departamento de Farmácia, Faculdade de Ciências Farmacêuticas, Universidade de São Paulo, Av. Lineu Prestes, 580, Cidade Universitária, São Paulo, SP, 05508-000, Brazil

[b] Centro de Biotecnologia, Universidade Federal do Rio Grande do Sul, Av. Bento Gonçalves, 9500, Porto Alegre, RS, 91500-970, Brazil

[c] Departamento de Bioquímica, Instituto de Química, Universidade de São Paulo, Av. Prof. Lineu Prestes, 748, São Paulo, SP, 05508-000, Brazil

[d] Centre for Molecular Informatics, Department of Chemistry, University of Cambridge, Cambridge, CB2 1EW, U. K.

[e] Departamento de Ciências Farmacêuticas, Faculdade de Ciências Farmacêuticas de Ribeirão Preto, Universidade de São Paulo, Av. Café, s/n, Ribeirão Preto, SP, 14040-903, Brazil

Sequences

HsSir2 - UniProt Q8IXJ6

TbSir2rp1 - NCBI AAC73004.1

Aligned sequences in file “emboss_needle_aligment_hSir2_TbSir2rp1.out”

DNA sequence of the expressed TbSir2rp1

5’-seq: CAT

ATGACAGAACCGAAGTTAGCAACCACGCACGTAGTGGGTGAACCCACCTT
CGAAGGACTGGCACGGTTCATTGAGCGAAACAACATCACCAAAATATTTGT
TATGGTGGGCGCAGGGATAAGCGTTGCAGCTGGAATCCCCGACTTCCGCT
CTCCCCACACCGGCTTGTACGCTAAACTCAGTCGCTACAATCTCAACTCAC
CGGAGGACGCCTTCTCACTCCCTCTCTTGCGTCAACAACCAAGTGTGTTTT
ACAACATTCTGATGGATATGGACCTCTGGCCCGGGAAGTATTGTCCTACGA
CGGTTCACCACTTTATCAGTCTACTCGCCAAGAAGGGCATGTTATTATGCT
GTTGTACGCAGAACATAGACGGGTTGGAACGCGCCTGCGGAATTCCAGAG
TCTTTACTAGTTGAAGCCCATGGTTCCTTCTCTTCCGCATCATGTGTTGACT
GTCACGCGAAATATGACATCAACATCGCGAGGGCGGAGACAAGGGCTGG
AAAAGTGCCTCATTGCAATCAATGTGGTGGTATAGTGAAACCCGACGTGGT
TTTCTTTGGCGAGAATCTCCCGGAGGCGTTTTTTAACGTAGCGGGACTCAT
TGAGGAAACGGAATTGCTGCTTATTTTGGGAACCTCACTTCAAGTCCACCC
ATTTGCCGACCTTGCGCTCATGGTGCCCTCTGACGTGCCACGAGTGTTGT
TTAACTTGGAGCGTGTGGGCGGGAGGATGTTCCGCTTTCCTACGGACCGA
ACACCCAATTTCCGCGCCAGTTCCTATCGTCTCAGCACTGGAAATGGCAAT
GGCAGTAAAATTAGCAGTGGGGACAGCAGCAGCAGCAGCAGCGTCGACG
GGTATGACCAGTTTACGCTCGCAGAGAATGACGAGACGGGTGTGTTGCGT
GACATTTTCTTTCCTGGTGACTGTCAGGTGTCTGTTCGTTCCTTTGCTCAG
GCGTTGGGCTTCGGAGAGCAGCTTGACGCCTCTGTACGTGAGGGAAGGG
AAATATTTGAGCGCACTCGGCGTAGGGAAAAAGTCGTTGAGGGT

3’-seq: CTCGAG

Length: 1062 bp

Table S1. Secondary and Tertiary Structure Evaluation: QMEAN and WHATCHECK Results

Server	Assessment Tool	Score
**SAVES**	Verify3D	87.72%
**SAVES**	ERRAT	68.2274
**SAVES**	PROVE	7.50%
**SAVES**	PROCHECK_1	W
**SAVES**	PROCHECK_2	E
**SAVES**	PROCHECK_3	W
**SAVES**	PROCHECK_4	P
**SAVES**	PROCHECK_5	E
**SAVES**	PROCHECK_6	E
**SAVES**	PROCHECK_7	W
**SAVES**	PROCHECK_8	P
**SAVES**	PROCHECK_9	P
**SAVES**	WHATCHECK_1	E
**SAVES**	WHATCHECK_2	E
**SAVES**	WHATCHECK_3	P
**SAVES**	WHATCHECK_4	-
**SAVES**	WHATCHECK_5	P
**SAVES**	WHATCHECK_6	P
**SAVES**	WHATCHECK_7	P
**SAVES**	WHATCHECK_8	P
**SAVES**	WHATCHECK_9	P
**SAVES**	WHATCHECK_10	P
**SAVES**	WHATCHECK_11	P
**SAVES**	WHATCHECK_12	P
**SAVES**	WHATCHECK_13	P
**SAVES**	WHATCHECK_14	P
**SAVES**	WHATCHECK_15	P
**SAVES**	WHATCHECK_16	P
**SAVES**	WHATCHECK_17	P
**SAVES**	WHATCHECK_18	W
**SAVES**	WHATCHECK_19	P
**SAVES**	WHATCHECK_20	W
**SAVES**	WHATCHECK_21	W
**SAVES**	WHATCHECK_22	P
**SAVES**	WHATCHECK_23	P
**SAVES**	WHATCHECK_24	P
**SAVES**	WHATCHECK_25	P
**SAVES**	WHATCHECK_26	W
**SAVES**	WHATCHECK_27	W
**SAVES**	WHATCHECK_28	W
**SAVES**	WHATCHECK_29	P
**SAVES**	WHATCHECK_30	W

**SAVES**	WHATCHECK_31	P
**SAVES**	WHATCHECK_32	P
**SAVES**	WHATCHECK_33	P
**SAVES**	WHATCHECK_34	E
**SAVES**	WHATCHECK_35	W
**SAVES**	WHATCHECK_36	W
**SAVES**	WHATCHECK_37	W
**SAVES**	WHATCHECK_38	P
**SAVES**	WHATCHECK_39	P
**SAVES**	WHATCHECK_40	W
**SAVES**	WHATCHECK_41	E
**SAVES**	WHATCHECK_42	P
**SAVES**	WHATCHECK_43	E
**SAVES**	WHATCHECK_44	E
**SAVES**	WHATCHECK_45	P
**SAVES**	WHATCHECK_46	W
**SAVES**	WHATCHECK_47	W
**SAVES**	WHATCHECK_48	P
**Swissmodel**	QMEAN4 Value	-1.91

Table S2. Screening results for the complete fragment library

% Inhibition TbSir2rp1	SMILES	MW	LogP	HBD	HBA	nRot
99 ± 0.47	OC1=C(C)C2=C(N=C(N)S2)C3=CC=CC=C31	230	2.2	2	3	0
84 ± 2.3	CC1=CC=C(S(N/N=C/C2=CC=CS2)(=O)=O)C=C1	280	4.8	4	1	4
88 ± 14	OC1=C(C2=C(CC)NNC2=O)C=CC=N1	205	1.2	3	4	2
78 ± 2.3	CN(C)C(C=C1)=CC=C1/C=N/C2=NN=C(O2)C3=CC=CC=C3	292	5.6	0	5	4
94 ± 3.1	O=[N+](C(C=C1)=CC=C1N2CCNCC2)[O-]	207	1.7	1	4	2
60 ± 5.5	O=C1NC2=CC=CC=C2C=C1	145	2.2	1	1	0
61 ± 9.1	ClC1=NC=C(NN=C2)C2=C1	153	1.1	1	2	0
55 ± 4.8	O=C1CC2=CN=CC=C2CN1	148	-0.1	1	2	0
65 ± 1.7	CC1=CC=C(S(N(C)/N=C/C2=CC=CS2)(=O)=O)C=C1	294	4.2	4	0	4
56 ± 4.9	N=C(N)N/N=C/C1=CC=CS1	168	2.5	3	4	3
89 ± 31	NC1=C2C=CC=CC2=CC=C1	143	3.6	1	1	0
102 ± 5.9	O=C(/C=C/C1=C(Cl)C=CC=C1)C2=CC3=C(OCO3)C=C2	286	4.6	0	1	3
95 ± 4.9	OC1=C(NC2=CC=CC=C2)C(C3=CC=CC=C3C1=O)=O	265	5.7	2	4	2
71 ± 23	OC1=C(NC2=CC=C(F)C=C2)C(C3=CC=CC=C3C1=O)=O	283	5.4	2	5	2
44 ± 3.7	O=C(C1=C2C=CC=C1)C(OC)=CC2=O	188	2.2	0	3	1
47 ± 10	O=C(NC)C1=C(C(C)C)OC2=C1C=CC(N3CCCC3)=N2	287	2.3	1	4	4
35 ± 3.7	CS(NC1=CC=CC(C2=CC=CC=C2)=C1)(=O)=O	247	5.2	1	3	3
27 ± 0.91	NC1=CC=CC([N+]([O-])=O)=C1	138	1.7	1	3	1
44 ± 24	O=[N+](C1=CC=CC(C2=NCCO2)=C1)[O-]	192	2.2	0	4	2
31 ± 13	C/C(C1=CC=NC=C1)=N\NC(N)=O	178	0.6	3	2	3
23 ± 6.4	C[C@H](NC)[C@@H](C1=CC=CC=C1)O	165	2.5	2	2	3
37 ± 24	NNS(=O)(C1=CC=C(Cl)C=C1)=O	206	0.8	4	2	2
19 ± 6.0	O=C(NC(C1(CC)CC)=O)NC1=O	184	0.4	2	3	2
19 ± 9.9	ClC1=NC=C(N(C(C)=O)N=C2)C2=C1	195	0.7	0	3	1
8.2 ± 1.4	O=C1NC(C(C)=CN1[C@@H]2O[C@H](CO)[C@@H](N=[N+]=[N-])C2)=O	267	-0.9	2	6	3
13 ± 10	C1(C2=NN=C(SCC3=CC=CC=C3)N2)=CC=CC=C1	267	6.5	1	2	4
5.8 ± 3.4	O=C(CN1)N(CCNC(C)=O)C1=O	185	-1.2	2	3	4
2.6 ± 1.1	N/C(NC1=CC=CC=C1)=N/C2=CC=CC=C2	211	4.9	2	3	3
19 ± 17	CC1=C(C(O)=O)C2=C(O1)N=CC=C2	177	1.3	1	4	1
3.0 ± 1.7	O=C1CCSC2=NC(C3=CC=CC=C3)=NN21	231	3.1	0	3	1
2.7 ± 1.6	O=C(NC(C1(CC)C2=CC=CC=C2)=O)NC1=O	232	2.8	2	3	2
4.9 ± 4.7	CC(C)(OC1=CC=C(C2C(Cl)(Cl)C2)C=C1)C(O)=O	288	3.6	1	3	4
2.7 ± 2.6	N1(CC2)CCN2CC1	112	-0.5	0	2	0
1.8 ± 1.9	N/C(N)=N\S(=O)(C1=CC=C(N)C=C1)=O	214	-0.3	3	6	2
4.6 ± 5.2	O=C(CN1)N(CCNC(C)=O)C1=O	143	-1.5	2	3	2
-2.1 ± 0.21	CC(C1=CC=C(CC(C)C)C=C1)C(O)=O	206	5.3	1	2	4
2.1 ± 5.0	O=C(N)N1C2=CC=CC=C2C=CC3=CC=CC=C31	236	4.2	1	1	1
-0.80 ± 4.1	CS(NC1=CC=CC(C2=NC3=C(C=CC=C3)N2)=C1)(=O)=O	287	3.0	2	4	3
-2.1 ± 3.0	O=C(N1C)N(C)C2=C(NC=N2)C1=O	180	-0.4	1	3	0
-3.3 ± 1.9	CCOC1NC(C2=CC=CC=C2)=NN1	191	3.9	2	4	3
0.85 ± 6.5	O=C(N)N1C2=CC=CC=C2CC(C3=CC=CC=C31)=O	252	2.9	1	2	1
0.91 ± 7.3	O=C1NC(C2=CC=CC=C2)=NN1	161	2.8	2	2	1
-4.7 ± 2.3	O=S(C1=CC=C(N)C=C1)(N)=O	172	-0.2	2	4	1
-5.5 ± 1.5	CC(NC1=CC=C(OCC)C=C1)=O	179	2.0	1	2	4
-4.9 ± 2.4	O=C(C1=CC=NC=C1)NN	137	-0.6	2	3	2
-4.0 ± 4.1	O=C(O)[C@@H](N)CC1=CC=C(O)C(O)=C1	197	-2.1	4	5	3
-6.5 ± 2.2	O=C(C1=CN(CC)C2=C(C=CC(C)=N2)C1=O)O	232	2.5	1	5	2
19 ± 28	NC1=CC2=NC(C3=CC=CC(C(F)(F)F)=C3)=CN2C=N1	278	3.8	1	6	2
8.8 ± 19	ClC1=NC=C(N)C(C)=C1	142	1.0	1	2	0
17 ± 27	OC1=CC(O)=CC(O2)=C1C(C3=CC=CC=C3)=CC2=O	254	3.8	2	4	1
1.6 ± 13	COC1=NC=C(NC(C)=O)C(C)=C1	180	0.8	1	3	3
8.4 ± 20	CC1=CC=C(S(N2N=CC=C2)(=O)=O)C=C1	222	2.2	0	3	2
-10 ± 1.7	O=C(O)C1=CC=CC=C1O	138	2.3	2	3	1
-2.9 ± 10	CC1=C(C(OCC)=O)C2=C(O1)[N+]([O-])=CC=C2	221	1.3	0	4	3
7.7 ± 21	CC(NC1=CC=CC=C1)=O	135	2.3	1	1	2
-7.1 ± 9.1	CC1=C(C(OCC)=O)C2=C(O1)N=CC(Br)=C2	283	2.5	0	4	3
2.3 ± 22	O=C(NN)CC1=CN=CC=C1	151	-0.6	2	3	3
-11 ± 11	O=C(C1=NN([C@@H]2O[C@H](CO)[C@@H](O)[C@H]2O)C=N1)N	244	-3.0	4	7	3
4.0 ± 30	CC1=CC=C(S(N2N=CC3=C2C=CC=C3)(=O)=O)C=C1	272	3.8	0	3	2
-22 ± 5.8	O=S(N1C=CC=C1)(C2=CC=C(C)C=C2)=O	221	3.0	0	2	2
-35 ± 5.0	O=C1C2=CC=CN2CCN1	136	0.3	1	1	0
-29 ± 12	CC1=C(C(OCC)=O)C2=C(O1)N=CC=C2	205	2.0	0	4	3
-29 ± 18	CC1=C(C(OCC)=O)C2=C(O1)N=C(Cl)C=C2	239	2.5	0	4	3
52 ± 2.3	O=C1NC(C(C2=CC=CC=C2)(C3=CC=CC=C3)N1)=O	252	4.7	2	2	2
49 ± 0.31	O=C1NC2=C(C=CC=C2)C1=O	147	1.7	1	2	0
53 ± 5.2	NNS(C1=CC=C(C)C=C1)(=O)=O	186	1.2	4	2	2

48 ± 1.8	O=C(NNC1=CC=CC=C1)NNC2=CC=CC=C2	242	5.2	4	3	6
48 ± 4.1	NC1=CC=C(S(C2=CC=C(N)C=C2)(=O)=O)C=C1	248	1.2	2	4	2
56 ± 15	COC1=NC=C(NN=C2)C2=C1	149	0.7	1	3	1
50 ± 11	O=C(OCCN(CC)CC)C1=CC=C(N)C=C1	236	2.0	1	4	7
43 ± 5.2	O=C1C2=C(OC(C)(C)CC2)C3=CC=CC=C3C1=O	242	3.2	0	3	0
38 ± 1.7	C1(/C=N/C2=NN=CS2)=CC=C(SC3=CC=CC=C3)C=C1	297	5.2	3	0	4
37 ± 2.2	O=C1N(C)C2=CC=C(Cl)C=C2C(C3=CC=CC=C3)=NC1	284	3.9	0	2	1
39 ± 4.6	OC1=C(NC2=C(C)C=CC=C2C)C(C3=CC=CC=C3C1=O)=O	293	5.9	2	4	2
42 ± 8.5	NNC(/C=C/C1=CC=CC=C1)=O	162	2.7	2	2	3
44 ± 12	ClC1=CC=C(S(C2=CC=C(Cl)C=C2)(=O)=O)C=C1	286	4.0	2	0	2
41 ± 12	C1(/N=C/C2=CC=CC=C2)=NN=C(CC3=CC=CC=C3)O1	263	7.4	0	4	4
32 ± 8.5	O=C(/C=C/C1=CC=C(Cl)C=C1)C2=CC=CS2	248	4.4	0	3	3
25 ± 4.0	OC(CNC(C)C)COC1=C2C=CC=CC2=CC=C1	259	4.1	2	3	6
22 ± 1.5	O=C(C1=NC=CN=C1)N	123	-1.1	1	3	1
33 ± 14	O=C(C1=C2OC(C)(C)CC1)C3=CC=CC=C3C2=O	242	3.2	0	3	0
22 ± 5.0	OC1[C@H](C(C)C)CC[C@@H](C)C1	156	3.2	1	1	1
24 ± 7.9	OCC1=CC2=C(NC(CNC(C)C)CC2)C=C1[N+]([O-])=O	279	1.8	3	5	5
16 ± 0.61	NC1=NN=C(O1)C2=CC=CC=C2	161	2.6	1	4	1
17 ± 4.4	O=C(N1)N(C)C2=C(N(C)C=N2)C1=O	180	-0.8	1	3	0
14 ± 1.5	O=C(N1C)N(C)C2=C(N(C)C=N2)C1=O	194	-0.6	0	3	0
12 ± 1.4	O=C(OCC)C1=CC=C(N)C=C1	165	1.7	1	3	3
14 ± 3.1	CC1=NC=C([N+]([O-])=O)N1CCO	171	0.0	1	4	3
10 ± 1.4	C12=NC(C3=CC=CC=C3)CN1CCS2	204	3.9	0	2	1
9.4 ± 1.0	SC1=NN=C(CC2=CC=CC=C2)S1	208	4.4	0	2	2

Table S3. Biological results for the sulfonyl-hydrazone series screened against TbSir2

Compound	Structure	IC_{50} or % inhibition at 100 µM	Compound	Structure	IC_{50} or % inhibition at 100 µM
2	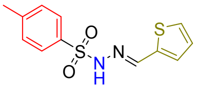	77.9 µM	2.13	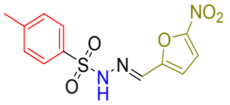	40.6 ± 13.6%
2.1	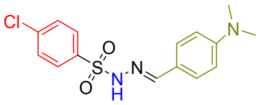	58.7 ± 3.1%	2.14	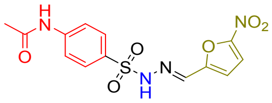	40.1 ± 2.7%
2.2	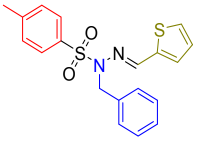	54.2± 1.0%	2.15	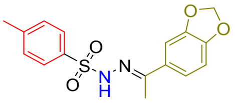	32.3 ± 1.7%
2.3	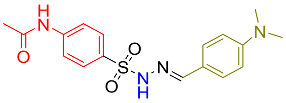	49.8± 2.4%	2.16	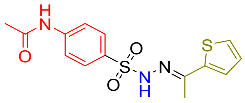	31.1 ± 0.7%
2.4	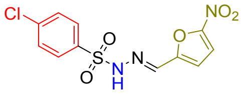	49.2± 10.5%	2.17	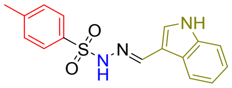	31.0 ± 0.9%
2.5	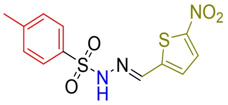	48.6± 7.5%	2.18	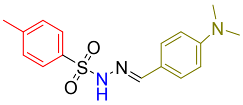	30.2 ± 5.6%
2.6	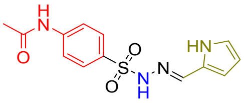	46.8± 3.6%	2.19	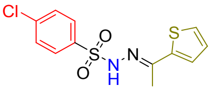	24.9 ± 2.8 %
2.7	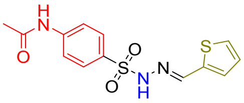	46.2± 0.7%	2.20	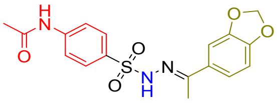	23.0 ± 1.5%
2.8	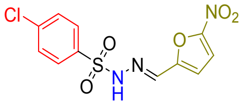	44.3± 4.6%	2.21	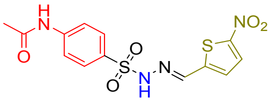	4.6 ± 1.1%
2.9	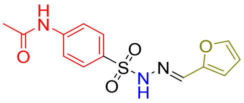	44.2± 2.5%	2.22	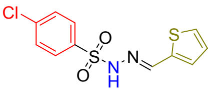	4.0 ± 6.0%
2.10	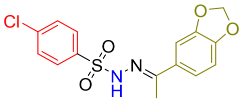	43.6± 3.4%	sulfamethazine	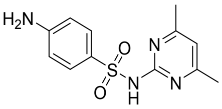	Inconc.[a]
2.11	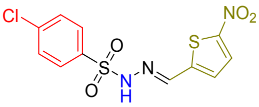	41.2± 3.0%	sulfamethoxazole	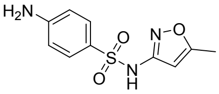	2.0 ± 5.3%

2.12	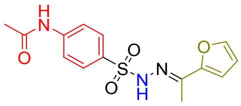	41± 6.4%	[a]Inconclusive.

Figure S1. Screening of Experimental Conditions for TbSir2rp1 Fragment-Based Screening

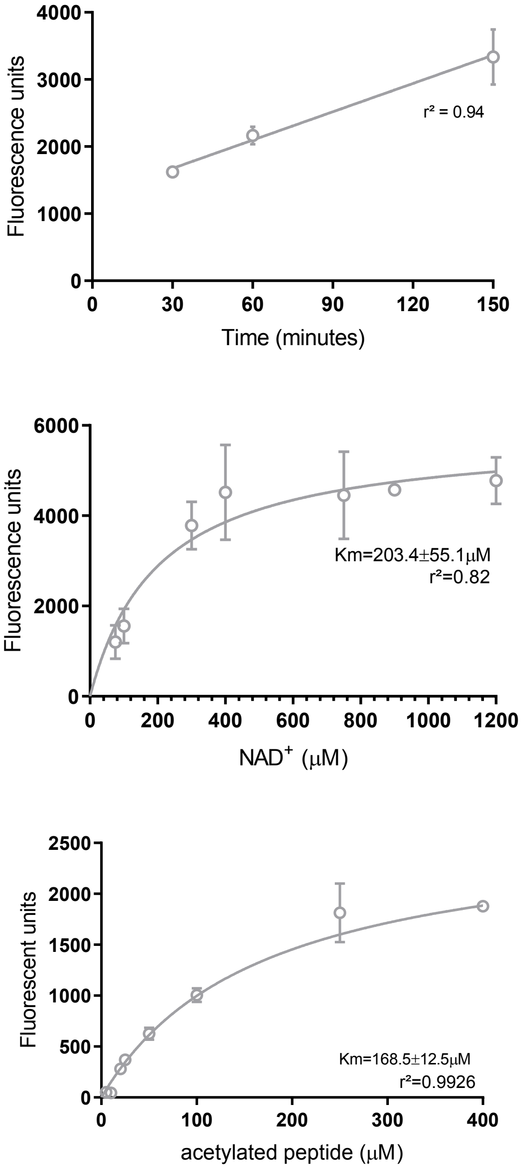

Supplement: Supplementary file 1 — ao4c09231_si_001.pdf [file ao4c09231_si_001.pdf]
